# Eudaimonic Well-Being of Italian Young Adults during the COVID-19 Pandemic: Predictive and Mediating Roles of Fear of Death and Psychological Inflexibility

**DOI:** 10.3390/ijerph20115960

**Published:** 2023-05-26

**Authors:** Vincenzo Calvo, Chiara Masaro, Chiara Fusco, Camilla Pellicelli, Simona Ghedin, Cristina Marogna

**Affiliations:** 1 Department of Philosophy, Sociology, Pedagogy, and Applied Psychology, University of Padova, 35131 Padua, Italy; 2 Department of Psychology, University of Milano-Bicocca, 20126 Milan, Italy; 3 Servizio per le Dipendenze ASL Roma 6, 00041 Anzio, Italy

**Keywords:** eudaimonic well-being, psychological inflexibility, fear of death, young adults

## Abstract

The literature has widely acknowledged the impact of the COVID-19 pandemic on the mental health of young adults. Despite extensive research, eudaimonic well-being, which focuses on self-knowledge and self-realization, has been scarcely investigated. This cross-sectional study aimed to add knowledge on the eudaimonic well-being of young adults one year after the outbreak of the COVID-19 pandemic, verifying its potential linkages with fear of death and psychological inflexibility. A total of 317 young Italian adults (18–34 years), recruited through a chain sampling method, completed measures of psychological inflexibility, fear of death, and eudaimonic well-being included in an online survey. The study’s hypotheses were tested with multivariate multiple regression and mediational analyses. Results showed that psychological inflexibility was negatively associated with all the dimensions of well-being, while fear of the death of others was associated with autonomy, environmental mastery, and self-acceptance. Furthermore, in the association between fear of death and well-being, the mediation role of psychological inflexibility was verified. These results contribute to the extant literature on the factors associated with eudaimonic well-being, providing clinical insights into the work with young adults within challenging times.

## 1. Introduction

### 1.1. Young Adults during the COVID-19 Pandemic

During the past three years, many scholars have investigated the psychological consequences of the COVID-19 pandemic, which broke out in Wuhan, China, in late 2019 and spread rapidly around the world [1,2,3].

In a context of isolation, fear of the physical consequences of the contagion, and uncertainty about the future [4], the literature has shown that young adults are an at-risk population from a psychological perspective [5].

Several studies have documented the association between younger age and lower levels of mental health during the pandemic [6,7]. In particular, young adults have been found to experience high rates of loneliness, depressive and anxious symptoms, stress, and sleep problems [7,8,9,10].

As highlighted by Stroud and Gutman [11], young adults have reported damaging effects of the COVID-19 pandemic on their mental health, despite their lower risk of severe COVID-related consequences for physical health. As scholars in this field pointed out, young adults were transitioning through a critical life phase at the time of the outbreak and, therefore, were particularly vulnerable compared to other social groups [5,11,12]. Young adulthood entails multiple transitions in education, employment, living arrangements, and close relationships. These transitions are normative and lead to young adults’ psychological development. At the same time, this challenging life phase also puts psychological well-being at risk [11,13].

The pandemic negatively affected labor market opportunities, especially for new entries, socialization, and learning occasions with friends and within remote educational pathways [6,14]. Although young adults could maintain social relationships through social networks [5], the literature has shown that engagement with social networks as an information source could be related to young adults’ higher stress levels during the pandemic [15,16].

### 1.2. Italian Young Adults’ Psychological Well-Being during the Pandemic

Italy was heavily affected by COVID-19 [17,18]. After China, it was the first country to adopt a national lockdown as a measure to contain the spread of the virus [3], announced on 8 March, only a few weeks after Italy’s first official COVID-19 case was detected at the end of February 2020. Staying at home was mandatory, while the media constantly documented the increase in positive cases. As Favieri et al. [4] pointed out, the Italian population was deeply affected by the sight of trucks carrying the coffins of the victims from the town of Bergamo (in northern Italy) to other Italian regions, as the death toll exceeded the capacity of the town’s cemeteries.

Scholars investigating the psychological impact of the pandemic on the Italian population have widely reported the psychological vulnerability of young adults in the context of the COVID-19 pandemic [3,16,19]. Rossi et al. [9] studied the mental health outcomes of the Italian adult population after the outbreak, showing that younger age was associated with post-traumatic stress symptoms, depression, anxiety, insomnia, high perceived stress, and adjustment disorder. In this direction, Pompili et al. [20] documented Italian young adults’ vulnerability to disordered alcohol and food behaviors during the lockdown. Concurrently, Parola et al. [21] reported increased levels of depression and anxiety in young Italian adults during the lockdown, in line with other studies supporting the likelihood among the younger Italian population of developing depressive and anxiety symptoms and stress during the pandemic [15,19,22].

Despite the literature on the psychological impact of the COVID-19 pandemic suggesting profound and long-lasting consequences related to lockdown measures and contagion restrictions [7], the attention to psychological well-being during subsequent pandemic phases seems limited [3,23]. Focusing on these phases may be particularly relevant to the Italian population, considering the restrictions implemented in Italy to face the consecutive waves of COVID-19 contagion. Unlike other countries that employed longer permanent closing strategies, the Italian government adopted the alternation of opening and closing regionally administered measures [15]. This strategy was held from November 2020 until the end of the sanitary emergency, declared on 31 March 2022, over two years later. During this time, Busetta et al. [15] registered increased anxiety levels among Italian university students when comparing the measures collected during the first lockdown (March 2020) and those obtained one year later, between March and April 2021. These findings can also be linked to the uncertainty and ambiguity induced by the Italian prolonged region-specific restrictions [15,18].

As some authors outlined [23,24,25], existing knowledge on the psychological impact of COVID-19 has predominantly focused on hedonic-related psychological issues, such as symptoms of anxiety and depression [16]. It is important, however, to distinguish two different domains of psychological well-being: ‘hedonic’ and ‘eudaimonic’ [26]. The former entails the presence of positive psychological states and the absence of negative ones. More specifically, hedonic well-being concerns attaining pleasure and happiness and avoiding pain [27,28]. On the other hand, eudaimonic well-being refers to humans’ optimal psychological functioning, achievable through realizing one’s potential and authentic self [29]. It focuses on existential qualities of psychological well-being, such as human growth, self-realization, and life meaning [30,31]. Since eudaimonic well-being relates to one’s pursuit of self-realization and attribution of meaning to life when confronting the world’s adversity [31], it is a crucial aspect of young adults’ psychological well-being, in particular during an existentially challenging historical moment such as the COVID-19 crisis, that deserves more investigation.

### 1.3. Fear of Death, Psychological Inflexibility, and Eudaimonic Psychological Well-Being during the Pandemic

Menzies and Menzies [32] highlighted the relevance of Terror Management Theory (TMT; [33,34]) to understand the psychological impact of the COVID-19 pandemic. TMT suggests that awareness of mortality produces the terror that encompasses the human condition [32]. According to TMT, the fear of death drives humans’ behaviors in an attempt to cope with this existential terror. The assumptions of TMT have been extensively studied and supported by laboratory research in which people were exposed to mortality-salient primes. These primes make salient to people their mortal condition and evoke the terror related to the awareness of one’s mortality [32,35].

Scholars suggested that the COVID-19 pandemic could have increased people’s fear of death [35,36], as the outbreak worked as a mortality prime in real life [32]. Curșeu et al. [37] showed that death anxiety predicted COVID-19 anxiety and negative mood during the pandemic; furthermore, scholars pointed out the positive relationship between individuals’ fear of death and psychological distress [38], depression [39], and anxious symptomatology [40].

Similarly, fear of death might also have influenced eudaimonic well-being during the pandemic. However, despite the relevance of such an argument, studies in this field are still scarce, and they entirely overlook young adults. To our knowledge, the relationship between fear of death and eudaimonic well-being was explicitly investigated only by Zhao et al. [41]. The authors outlined a significant negative relationship between death anxiety and the hedonic well-being of Chinese front-line medical staff; concurrently, no relationship was found between death anxiety and eudaimonic well-being. However, eudaimonic well-being was assessed using a scale focusing only on a specific aspect of well-being (i.e., meaning in life), whereas other relevant key components of eudaimonic well-being were neglected [31]. Therefore, further studies on the role of factors potentially clarifying the relationship between fear of death and eudaimonic well-being are necessary to increase our understanding of the psychological impact of the pandemic.

As highlighted by Visintin and Tasso [42], during the pandemic, media and political discourse suggested that people with debilitating diseases or older people had a higher likelihood of severe complications in case of COVID-19 infection. In Italy, the official communications during the early stages of the pandemic underlined that COVID-19 was more likely to severely affect only vulnerable people, specifically older adults, and sick people, leading to the diffusion of age-related rhetoric by Italian media about COVID vulnerability [43,44].

Along with representing older people as the most vulnerable population [3], national media discourse misleadingly contributed to conveying that “only older adults, not the young” were an at-risk population for COVID-19 infection [43] (p. 535). Along this line, Sjölander-Lindqvist et al. [45] found that, within the Italian context, the communication of then Prime Minister Conte aimed at underlining the importance of an intergenerational building of meaning around older people’s deaths in Italy, encouraging Italians to adopt preventive behaviors and social distancing to protect “especially the health of our grandparents” [42] (p. 2).

Building on these premises, it is likely that the mortality-salient pandemic context could have contributed to higher levels of fear of the death of others than of one’s death among young Italian adults. In this direction, it appears arguable to distinguish two dimensions of fear of death—of self and others—to investigate the psychological well-being of young Italian adults during the pandemic as a contextually oriented choice that could further enlighten our understanding.

The literature highlighted that psychological inflexibility was a risk factor during the COVID-19 crisis [46]. The construct of psychological inflexibility is rooted in the framework of Acceptance and Commitment Therapy (ACT [47]). ACT aims to increase people’s ability to seize situational opportunities. To this end, ACT promotes people’s flexibility, allowing them to behave consistently with personal values and long-term goals [48,49]. Within this framework, psychological inflexibility refers to the inability to persist or change behaviors consistently and effectively with one’s goals. This incapacity reflects the rigid dominance of individuals’ psychological reactions in guiding action over personal values and contextual events [47,48]. Psychological inflexibility has been conceptualized as the result of two processes: cognitive fusion, i.e., perceiving self-descriptive and evaluative thoughts as literally real, and experiential avoidance, i.e., the tendency to avoid experiencing undesirable internal events [48,49]. In their study on suicidal risk during the pandemic, Crasta et al. [46] showed that psychological inflexibility seems to exacerbate COVID-19-related stress. The authors acknowledged that psychological inflexibility represents a risk factor for psychological well-being, supporting the conceptualization of psychological inflexibility as a maladaptive psychological factor [27] associated with the likelihood of developing depressive, anxiety, and stress symptoms during the pandemic [50].

The literature has supported the potentially negative effect of psychological inflexibility on eudaimonic well-being [27,51]. To our knowledge, however, only one study investigated the association between psychological inflexibility and eudaimonic well-being within the pandemic context [52]. The research showed, as expected, a significant negative association between eudaimonic well-being and inflexibility in urban Indian mothers [52].

In the attempt to identify potential factors that could mitigate the impact of the COVID-19 pandemic on the well-being of individuals, Arslan et al. [53] found that psychological inflexibility could play a significant role in mediating the adverse effects of coronavirus on depression, anxiety, and somatization among young adults. This result led the authors to claim that, from a theoretical perspective, COVID-related stress may play a predictive role in increasing people’s psychological inflexibility [53], in line with other studies suggesting the cruciality of psychological inflexibility in understanding individuals’ coping and adaptation within the adverse situations brought about by the COVID-19 pandemic [54,55].

### 1.4. The Present Study

The COVID-19 pandemic has represented a collective trauma that undermined people’s expectation of a known and trustworthy world [56,57]. 

Young adults have been dealing with a highly meaning-demanding context, with less certainty over their future, and faced this during a multiple-transition life phase, in which one’s life plans, pursuits, and beliefs are challenged [13]. Italy was one of the most severely affected countries in Europe. In addition, during the subsequent pandemic phases, the contagion containment strategy implemented in Italy appeared to have put the Italian population at a higher psychological risk [15]. To our knowledge, only one study [23] investigated the eudaimonic well-being of young Italian adults in the subsequent phases of the pandemic. The authors considered its association with rates of non-suicidal self-injury episodes in the aftermath of the outbreak from November 2020 to January 2021. 

The present study aimed to add knowledge to the eudaimonic well-being of young Italian adults one year after the start of the COVID-19 outbreak investigating the relationship between fear of death, psychological inflexibility, and eudaimonic well-being. The recent Self- vs. External Regulatory Behavior Theory (SR-ER Theory) [58,59] can contribute to formulating predictions about the direction of the relationships among these three constructs. According to the SR-ER theoretical model, variability in human behavioral regulation depends on the combination of personal and contextual factors. Personal factors include three different types or levels of behavioral self-regulation, ranging from the more adaptive and positively proactive behavioral level—named the Self-Regulation Behavior Type (SR)—to the less adaptive and negatively proactive behavioral level, the Dys-Regulation Behavior Type (DR), with an intermediated, neutral behavioral level, named the Non-Regulation Behavior Type (NR). 

Similarly, contextual factors are categorized into three levels of external regulation, External Regulation (ER), External Non-Regulation (ENR), and External Dys-Regulation, (EDR) and are considered influential in determining the variability of the person’s self-regulation, provoking or fostering SR, NR, and DR behaviors, respectively. The SR-ER theoretical model advocates that the interaction between the person’s SR-NR-DR self-regulatory behavioral levels and the contextual ER-ENR-EDR levels is predictive of various adaptive and maladaptive variables, such as psychological well-being and emotional dysregulation [59,60]. In particular, the combination of external dysregulation and personal dysregulation is thought to have the worst impact on the person’s psychological regulation. This has been observed in different areas of behavior, both in the clinical and health fields [58,59].

According to this theoretical framework, the pandemic can be considered a natural dysregulation contextual factor, functioning as an External Dys-Regulation variable that negatively impacts the person’s adaptation and well-being. Similarly, in the SR-ER theoretical model, higher levels of fear of death and psychological inflexibility can be viewed as dys-regulatory behaviors (DR) that are expected to reduce an individual’s adaptation and eudaimonic well-being. Therefore, in line with the assumptions of the SR-ER theoretical model, we hypothesized that psychological inflexibility and fear of death were negatively associated with young adults’ eudaimonic well-being. Moreover, based on the literature supporting the role of psychological inflexibility in mediating the negative effects of COVID-19-related stressors on psychological well-being [53,54], we hypothesized that psychological inflexibility could mediate the potential negative effect of the fear of death elicited by the pandemic [61] on young adults’ eudaimonic well-being.

In detail, the goal of the current study was threefold. First, we aimed to verify whether psychological inflexibility was negatively associated with the eudaimonic well-being of young adults one year after the onset of the pandemic. The second aim was to investigate the association between fear of death and eudaimonic well-being and, third, to verify the mediational role of psychological inflexibility in this association.

## 2. Materials and Methods

### 2.1. Participants

A convenience sample of 317 young Italian adults participated in the study. Their ages ranged from 18 to 34 years, with a mean age of 25.29 (SD = 3.60) years; 231 were females (72.9%), and 86 were males (27.1%). Their mean level of education was 15.5 years (range: 8–21 years). Levels of education included lower secondary education (1.6%), upper secondary education (32.4%), college and university education (63.5%), and postgraduate degree (2.5%). A total of 92 of the participants were employed (29.1%), 29 were unemployed (9.2%), and 195 were students (61.7%).

### 2.2. Procedure

Young Italian adults were recruited via social media announcements (Facebook, Instagram, etc.) inviting them to participate in a study about psychological well-being during the pandemic. An anonymous Italian-administered online survey was created and distributed via social media and messenger platforms (Facebook, Instagram, WhatsApp Messenger) using a chain sampling method. The study announcements included a description of the research and an internet link to the online survey that people could autonomously click to access the research protocol. To participate, the survey preliminarily required the respondents’ consent for their voluntary and anonymous participation and data processing. The survey was intended to collect socio-demographic and pandemic-related information about the participants and included validated self-report questionnaires measuring their eudaimonic well-being, psychological inflexibility, and fear of death.

The inclusion criteria were being a young adult between the ages of 18 and 34; being Italian; and having lived in Italy between March and May 2020.

The study was approved by the Ethics Committee for Psychological Research of the University of Padova (protocol id. 3693). The requirement for written consent was waived by the ethics committee because of the anonymous data collection.

Data were collected from 20 to 28 March 2021, about one year after the start of the onset of the COVID-19 pandemic and in the aftermath of a new surge of infections affecting Italy. A total number of 507 individuals responded to the survey, but 172 records were discarded because respondents were older than 34 years (*n* = 159), their age was missing (*n* = 8), socio-demographic data were lacking (*n* = 5), or they were not Italian and/or not living in Italy between March and May 2020 (*n* = 18), thus resulting in a final sample of 317 participants.

### 2.3. Measures

#### 2.3.1. Eudaimonic Well-Being

The Psychological Well-Being Scale (PWB) [29], in its 84-item Italian-validated version [62], was used to assess eudaimonic well-being. This instrument assesses the dimensions of autonomy, environmental mastery, personal growth, positive relations with others, purpose in life, and self-acceptance on a 6-point Likert scale. Answers range from 1 (strongly disagree) to 6 (strongly agree), to items such as: ‘I have a sense of direction and purpose in life’. Higher total scores of the subscales point to higher well-being. PWB’s reliability ad validity is widely acknowledged, and its Italian version showed satisfactory psychometric properties [63]. In the present study, the PWB subscale scores showed very good internal consistency (Cronbach’s α autonomy = 0.85, environmental mastery = 0.86, personal growth = 0.83, positive relations with others = 0.88, purpose in life = 0.86, and self-acceptance = 0.90).

#### 2.3.2. Psychological Inflexibility

We used the 7-item Italian version of the Acceptance and Action Questionnaire-II (AAQ-II) [48,64] to measure psychological inflexibility. The AAQ-II is scored on a 7-point scale ranging from 1 (not at all/never true) to 7 (very/always true). An example item is ‘I worry about being unable to control my worries and feelings’. The AAQ-II operationalizes the construct of psychological inflexibility as experiential avoidance (i.e., unwillingness to experience unwanted emotions and thoughts), rigid psychological reactions in guiding action, and the inability to be in the present moment [48]. Higher overall scores (range: 7 to 49) indicate greater psychological inflexibility. The alpha coefficient in the present study was high (α = 0.90).

#### 2.3.3. Fear of Death

The Italian version of the revised Collett–Lester Fear of Death Scale (CL-FODS) [65,66] was used to measure the fear of death of the participants. It is the third version of the first Collett–Lester Fear of Death and Dying Scale (Collett and Lester, 1969). Respondents are asked to rate how much they are disturbed or made anxious by 28 aspects of death and dying concerning the self and others, using a 5-point Likert scale (0, not to 5, very). These items contribute to four subscales: fear of death of self, fear of dying of self, fear of death of others, and fear of dying of others. Sample elements include ‘The pain involved in dying’ referring to ‘Your own dying’ and ‘Losing someone close to you’ referring to ‘The death of others’. Consistency coefficients in the current study were adequate, with all Cronbach’s alphas greater than 0.75 (death of self = 0.82, dying of self = 0.81, death of others = 0.76, and dying of others = 0.81). For the purposes of this study, we summarized the first two subscales to provide a total score for ‘fear of death and dying of self’ and the second two subscales for the ‘fear of death and dying of others’.

### 2.4. Data Analyses

Descriptive statistics and Pearson’s correlations among study variables were calculated. 

Extant literature suggests that age and gender [67], as well as employment status and employment experiences [68], are associated with or impact psychological well-being in young adults. Therefore, the potential confounding effects of age, gender, and employment status (currently employed or not) were examined and controlled for in the subsequent analyses.

A multivariate regression was carried out to verify the first hypothesis, testing the degree to which psychological inflexibility (predictor) relates to the six subscales of psychological well-being (dependent variables), controlling for confounders.

To test the second hypothesis, concerning the association between fear of death and psychological well-being, we carried out a multivariate multiple regression using both fear of death and dying of self and fear of death and dying of the others as the predictors or independent variables, the PWB subscale as the dependent variables, controlling for confounders.

Lastly, we run mediational analyses to test our hypothesis. Fear of death was treated as the predictor variable (X), psychological inflexibility as the mediator (M), and the well-being subscales as the outcome (Y). Mediations were calculated following a regression-based approach using Process 3.5.2 [69], an SPSS macro add-in. Coefficient estimates of the direct and indirect effects of the models and their statistical significance were generated. The indirect effects of the mediation hypotheses were verified using the 95% percentile bootstrap confidence intervals (CIs), computed with 5000 bootstrap samples. Indirect effects with CIs not crossing zero were considered statistically significant [70].

## 3. Results

As preliminary analyses, we computed descriptive statistics and zero-order Pearson’s correlations among study variables, presented in Table 1.

Correlation analyses indicated that subscales of psychological well-being are significantly associated among them, in some instances with a large effect size (≥0.5) according to Cohen’s [71] conventions for statistical effects. As expected, psychological inflexibility was negatively associated with all subscales of psychological well-being (in three instances, the effect size was “large” and in three “medium”, i.e., ≥0.30). 

We verified the association between psychological well-being scales and socio-demographic characteristics of the participants that the literature indicates as potential confounders: age was significantly correlated with environmental mastery (*r* = 0.18, *p* = 0.001), purpose in life (*r* = 0.22, *p* < 0.001); gender was correlated with personal growth (*t* [315] = −2.63, *p* = 0.009); and being currently employed (no/yes) was associated with environmental mastery (*t* [314] = −6.48, *p* < 0.001), purpose in life (*t* [314] = −5.67, *p* < 0.001), and self-acceptance (*t* [314] = −3.34, *p* < 0.001) (*n* = 316 because one participant did not indicate the current employment status). Participants’ years of education were not significantly correlated with the well-being measures; therefore, it was not considered a confounder in the subsequent analyses.

We conducted a multivariate regression to verify whether psychological inflexibility predicted the six dimensions of psychological well-being, controlling for gender, age, and employment status (*n* = 316). A multivariate main effect was found (Pillai’s Trace = 0.587, *F*(6, 305) = 72.21, *p* < 0.001). Psychological inflexibility was negatively associated with all subscales of PWB: autonomy (*b* = −0.36, *t* = −8.42, *p* < 0.001), environmental mastery (*b* = −0.54, *t* = −14.25, *p* < 0.001), personal growth (*b* = −0.28, *t* = −8.70, *p* < 0.001), positive relations with others (*b* = −0.48, *t* = −9.42, *p* < 0.001), purpose in life (*b* = −0.55, *t* = −12.86, *p* < 0.001), and self-acceptance (*b* = −0.67, *t* = −18.73, *p* < 0.001).

To verify the second hypothesis regarding the predictive association between fear of death and dying and well-being, multivariate multiple regression was carried out. Fear of death and dying of self and fear of death and dying of others were treated as the predictors, the PWB subscale as the dependent variables, and the confounders as a covariate (age) or as fixed factors (gender, employment status; *n* = 316). Results showed a significant multivariate effect of fear of death and dying of others (Pillai’s Trace = 0.071, *F*(6, 304) = 3.90, *p* = 0.001), but not of fear of death and dying of self (Pillai’s Trace = 0.020, *F*(6, 304) = 1.05, *p* = 0.395).

In detail, fear of death and dying of self was not significantly associated with any dimensions of well-being (all univariate effects with *p* > 0.05). In other words, the multivariate multiple regression indicated that fear of death and dying of self was not significantly associated with autonomy, environmental mastery, personal growth, positive relations with others, purpose in life, and self-acceptance of young adults participating in the study. Fear of death and dying of others, instead, showed three significant univariate negative associations: higher levels of fear of death predicted lower autonomy (*b* = −0.22, *t* = −3.79, *p* < 0.001), lower environmental mastery (*b* = −0.20, *t* = −3.25, *p* = 0.001), and lower self-acceptance (*b* = −0.18, *t* = −2.75, *p* = 0.006).

In light of previous results, we tested the mediational role of psychological inflexibility in the association between fear of death and dying of others and autonomy, environmental mastery, and self-acceptance; we excluded from this analysis fear of death and dying of self and the other three PWB scales because they did not meet Step 1 in establishing mediation (i.e., the causal variable should be correlated with the outcome; [72]).

**Figure 1:**
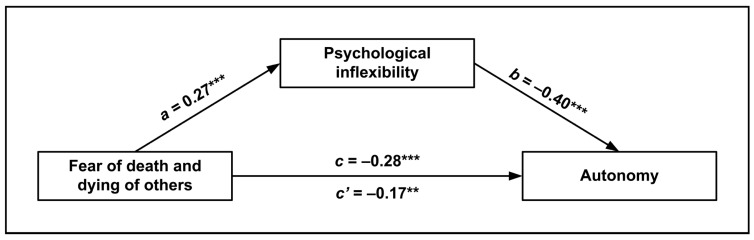
Mediation model of the relationship between fear of death and dying of others and autonomy, as mediated by psychological inflexibility. Standardized regression coefficients (*β*) are represented with solid pathways (** *p* < 0.01, *** *p* < 0.001). The *c* path is the total effect of fear of death of others on autonomy. The *c*’ path is the direct effect of fear of death of others on autonomy, estimated from the model when the mediator is present. Covariates are omitted from the figure for visual clarity.

The first mediation analysis included “fear of death and dying of others” as the predictor, psychological inflexibility as the mediator, and autonomy as the outcome, controlling for confounders. The mediation was significant overall, *F*(5, 310) = 16.68, *p* < 0.001, and accounted for 21% of the variance of autonomy. All the direct effects of the model were significant (Figure 1), as well as the indirect effect of fear of death of others on autonomy, via psychological inflexibility (*β* = −0.110, 95% CI [−0.167, −0.056]). Therefore, the first mediation hypothesis was established. As shown in Figure 1, fear of death and dying of others was positively associated with psychological inflexibility, which in turn was negatively linked with autonomy. Additionally, there was a negative direct association between fear of death and autonomy, indicating a partial mediation.

The next mediation analysis was carried out using the same predictor, mediator, and confounders of the previous, but treated environmental mastery as the outcome. The analysis was significant, *F*(5, 310) = 55.50, *p* < 0.001, and explained 47% of the variance. 

**Figure 2:**
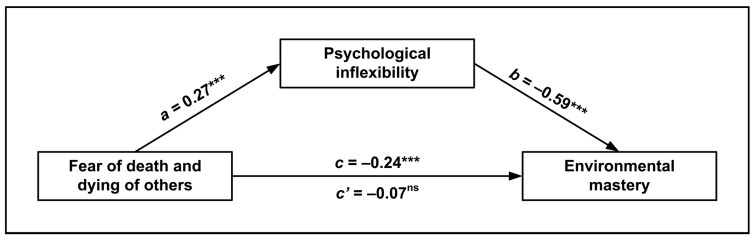
Mediation model of the relationship between fear of death and dying of others and environmental mastery, as mediated by psychological inflexibility. Standardized regression coefficients (*β*) are represented with solid pathways (ns = not significant; *** *p* < 0.001). The *c* path is the total effect of fear of death of others on environmental mastery. The *c*’ path is the direct effect of fear of death of others on environmental mastery, estimated from the model when the mediator is present. Covariates are omitted from the figure for visual clarity.

The indirect association of fear of death and dying of others with environmental mastery was significantly mediated by psychological inflexibility (*β* = −0.162, 95% CI [−0.239, −0.091]), showing a so-called complete mediation (Figure 2). Increasing levels of fear of death were associated with greater psychological inflexibility and lower environmental mastery.

**Figure 3:**
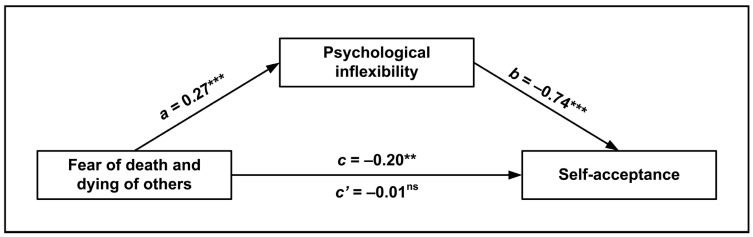
Mediation model of the relationship between fear of death and dying of others and self-acceptance, as mediated by psychological inflexibility. Standardized regression coefficients (*β*) are represented with solid pathways (ns = not significant; ** *p* < 0.01, *** *p* < 0.001). The *c* path is the total effect of fear of death of others on autonomy. The *c*’ path is the direct effect of fear of death of others on autonomy, estimated from the model when the mediator is present. Covariates are omitted from the figure for visual clarity.

The last mediation analysis involved the same measures, with self-acceptance as the dependent variable. Again, the overall analysis was significant, *F*(5, 310) = 75.12, *p* < 0.001, and the *R*^2^ = 0.55, indicating a high percentage of explained variance. The mediating role of psychological inflexibility was established (*β* = −0.204, 95% CI [−0.294, −0.109]) and the mediation was complete (*c* = −0.195, *p* = 0.001; *c*’ = 0.010, ns). Figure 3 reports the standardized effects emerging from the model. Once again, higher fear of death was associated with greater psychological inflexibility and, in turn, with lower self-acceptance.

## 4. Discussion

The general purpose of this cross-sectional study was to investigate eudaimonic well-being as related to psychological inflexibility and fear of death, two psychological factors that, according to the literature, could have had a significant role in influencing the well-being of young adults during the COVID-19 pandemic. Specifically, our study aimed to add knowledge on the psychological well-being of young Italian adults one year after the outbreak. The national and international literature documented young adults’ poor mental health during the pandemic [9]. Furthermore, in the aftermath of the perduring national lockdown from March to May 2020, the Italian government managed the subsequent contagion waves—from November 2021 to March 2022—implementing an alternation of opening and closing regionally administered measures, potentially putting the Italian population at a higher psychological risk due to this unstable strategy [15].

Regarding the study’s first aim, the analysis indicated that, as expected from the literature and the SR-ER theoretical model [58,59], psychological inflexibility was negatively associated with the eudaimonic well-being of young adults participating in the study. This result is in line with the SR-ER theoretical model that postulates that dys-regulatory behaviors (DR), such as high levels of psychological inflexibility, can reduce the person’s adaptation and psychological well-being. Higher levels of inflexibility were statistically predictive of lower scores in all six core components of Riff’s [30] model of eudaimonic well-being, including autonomy, environmental mastery, personal growth, positive relations with others, purpose in life, and self-acceptance.

To summarize Riff’s model, autonomy reflects the capacity of the human being to be independent, self-regulating, and self-determining; environmental mastery indicates the individual’s ability to gain mastery of the surrounding environment, a key component for positive psychological functioning; personal growth is related to the achievement of self-realization; positive relations with others reflect the depth of connections the person has with significant others; purpose in life refers to the extent to which respondents feel their lives have meaning, purpose, and direction; and finally, self-acceptance is the knowledge and acceptance of self, including awareness of personal limitations [31,73]. The model is based on a philosophic formulation of the concept of well-being, rooted in the ideas of Aristotle and ancient Greeks: “first, to know yourself, and second, to become what you are” [31] (p. 11). Both these sides of eudaimonic well-being—self-knowledge and self-realization—seem to be hindered by psychological inflexibility in young adults during the pandemic. These results are in line with earlier studies conducted both before the onset and during the pandemic, indicating that psychological inflexibility is negatively associated with eudaimonic well-being in adulthood [27,51,52]. Taken together, our results and previous literature seem to corroborate the hypothesis that psychological inflexibility is a maladaptive cognitive and emotion regulation strategy associated with a worse quality of life and reduced psychological well-being in adults and young adults. Psychological inflexibility can be described as a particularly problematic response style [74], characterized by a rigid pattern of reactivity and an inability to adaptively modify one’s behavior in pursuing one’s goals; therefore, this mental rigidness, as hypothesized by the ACT framework [75], can undermine the pursuit of self-knowledge and self-realization.

This can be a crucial setback in young adulthood, a phase of personal development that represents the long transition from adolescence to adulthood. Young adults have been facing psychologically challenging transitions characterizing this developmental life phase [13] while the pandemic was undermining labor and socialization opportunities [6,14] with an overall detrimental impact on people’s trust in the world [56,57].

The study’s second aim was to verify the hypothesis that fear of death was associated with eudaimonic well-being in young adults during the pandemic. We expected that higher levels of fear of death were associated with lessened well-being, as postulated by the SR-ER theoretical model [58,59]. The statistical analysis partially confirmed this expectation. More in detail, the fear of death and dying of others proved to be negatively associated with three key components of eudaimonic well-being out of six—autonomy, environmental mastery, and self-acceptance—but not with personal growth, positive relations with others, and purpose in life. On the contrary, the fear of death and dying of the self was not associated with eudaimonic well-being. In this direction, our understanding of eudaimonic well-being in the pandemic context could benefit by considering the multidimensional nature of the construct of fear of death, including both fear of one’s own death and fear of the death of others [76]. This distinction seems crucial in reference to young adults who, in the pandemic context, did not represent an at-risk population from a physical health perspective [9]. Our results are consistent with this interpretation and add knowledge regarding the peculiarities of the Italian context, where media discourses encouraged young adults to adopt preventive behaviors and restrict socialization occasions—during the pandemic phases—to prevent contraction of the virus for other people potentially belonging to at-risk populations because of their age or the presence of former pathologies. In this scenario, our result seems to suggest that, while the pandemic did not represent a direct threat to young Italian adults’ lives, it seemed to have threatened their fear for the physical health of potentially at-risk people, increasing the levels of their fear of death and dying of others, which, ultimately, had a negative impact on certain dimensions of their eudaimonic well-being. At the same time, statistical analyses identified significant negative associations between fear of death of self and dimensions of eudaimonic well-being. Therefore, our interpretation needs further investigation to understand the role of fear of death of self and others on young adults’ well-being, for example, by testing other mortality-salient conditions. We argue that further investigation in this direction could be relevant to add knowledge to the extant literature on the differences in death anxiety levels at different life cycle stages.

Interestingly, the significant associations between fear of death and dying of others and eudaimonic well-being involved three specific domains of well-being particularly important for young adults during the pandemic. First, individuals with higher levels of fear of death of other persons showed lower autonomy. According to Ryff’s [30] model, individuals with low autonomy are likely to exhibit scarce willpower and independence; furthermore, they are preoccupied with other’s expectations and evaluations and tend to rely predominantly on the judgments of others for making decisions [77]. During the pandemic, it is likely that young adults experienced heightened worry for the well-being of important family members, relatives, or friends who were at a higher risk of health complications from COVID-19. This concern may have been influenced by the increased awareness of mortality and reminders of death during the pandemic, potentially leading to an amplified fear of their own mortality [78]. As a result, the pandemic’s intensified focus on mortality could have augmented the psychological importance of significant others, whom young adults typically depend on, consequently reducing their feelings of autonomy and independence. 

Similarly, the fear of death of others due to the pandemic may have diminished the sense of control over their external world, a typical characteristic of people with low levels of environmental mastery and lacking a solid sense of competence in managing the environment. Environmental mastery is defined as a sense of control that includes feelings of efficacy in dealing with environments in general. This result is not surprising because young Italian adults, at the time of data collection, had already experienced long periods of lockdown, including isolation from the external environment, and repeated movement restrictions, factors that are thought to engender significant lockdown-related stress among this population [79]. Research has shown that sense of control is negatively associated with daily stress [80] and has suggested that the restrictions due to the pandemic and social distancing measures may have interfered with adults’ sense of control [81].

Lastly, the fear of death of others was associated with lower self-acceptance in young adults. Self-acceptance implies a benevolent attitude toward the self, acknowledging and accepting positive and negative individual qualities [77]. On the contrary, persons with low self-acceptance are likely to be unsatisfied with themselves, desiring or even struggling to be different. The mental well-being of young adults may have been significantly impacted during the pandemic by decreased levels of self-acceptance. Research indicates that individuals with higher self-esteem are better equipped to shield themselves from the adverse psychological effects of loneliness and fear stemming from COVID-19, such as anxiety and depression [82].

The third aim of this study was to verify the mediation role of psychological inflexibility in the association between fear of death and eudaimonic well-being. The mediation analyses exclusively targeted the three dimensions of eudaimonic well-being that were found to be associated with the fear of death and dying of others. Statistical analysis showed that psychological inflexibility significantly mediates the association between fear of death and dying of others in all the three eudaimonic dimensions examined: autonomy, environmental mastery, and self-acceptance. These findings suggest that inflexibility can be one of the mechanisms linking fear of death to eudaimonic well-being among young Italian adults during the pandemic. According to this result, an increased fear of death concerning significant others may have amplified young adult’s psychological inflexibility which, in turn, may have reduced their well-being. According to the ACT framework, inflexibility is a psychological factor encompassing various inflexibility processes, including cognitive fusion and experiential avoidance [75]. Experiential avoidance, in particular, has been defined as the phenomenon of attempting to avoid unpleasant private experiences (such as thoughts, feelings, emotions, sensations, etc.), which are expected to be excessively negative or distressing, through deliberate efforts to control, suppress, eliminate, or escape from them [75,83]. In some cases, a certain degree of experiential avoidance could function as a self-protective strategy to prevent consequences perceived as catastrophic [83]. In other cases, when too rigid and/or pervasive, experiential avoidance can be a pathogenic process [84], a toxic diathesis underlying several psychological vulnerabilities [84]. In the first place, the frequent use of experiential avoidance is exhausting for individuals and this aspect has spillover effects, including hindering well-being and interpersonal relationships [74]. Furthermore, although experiential avoidance can provide temporary relief to the person, it can have the paradoxical effect of amplifying those unwanted internal experiences that it was supposed to get rid of [74,85], engendering a vicious cycle that serves to exacerbate distress.

The pandemic has represented a longer-lasting mortality reminder; the elicitation of peoples’ awareness of mortality could have increased individual levels of fear of death and death anxiety [35]. Specifically, young adults could have experienced more experiential avoidance to face the fear of the death of significant others who, during the pandemic, were at a potentially physically higher risk, such as parents or grandparents, with the unwillingness to remain in contact with such painful thoughts and emotions in such a stressful and challenging situation. The increase in experiential avoidance, a fundamental process of psychological inflexibility, in turn, may have become a setback for their flourishing, hampering their eudaimonic well-being. 

As we have already mentioned, there is consistent literature supporting the negative link between psychological inflexibility and well-being [27,51,52], whereas studies focusing specifically on the association between fear of death and inflexibility processes are still very limited. Nonetheless, the findings of the only existing empirical study are coherent with the hypothesis that fear of death and psychological inflexibility might be positively associated. Gong et al. [86], indeed, have recently investigated this subject in patients with cancer, showing a significant positive correlation, with a large magnitude (i.e., >0.50), between experiential avoidance and death anxiety. Furthermore, research has highlighted the interplay between experiential avoidance, anxiety, and fear, showing that individuals who often employ experiential avoidance are more at risk for developing anxiety disorders [74], experiencing fear of intense emotions, and endorsing more frequent worry [87].

Taken together, these findings are compatible with the idea that inflexibility processes may mediate the relationship between fear of death and death anxiety and psychological well-being, but further research is needed.

The current study has several limitations that should be acknowledged. First, the study is cross-sectional and correlational and, therefore, it does not allow drawing inferences about the causal relationships between the variables investigated. Second, we did not collect any measures before the outbreak of the pandemic, such as measures assessing participants’ fear of death before the pandemic. Consequently, we have no concrete evidence that the pandemic has increased the fear of death due to the increased mortality salience and death awareness, as hypothesized by TMT. Third, we used self-report measures which are at risk for common method and common rater biases [88]. Moreover, data were collected with an online survey, which has some potential limitations concerning the self-selection of participants and their unknowability by the researcher [89,90]. Nonetheless, this method allowed the collection of an appreciable number of participants to verify the mediation hypothesis with enough statistical power while complying with the measures for the spread of the contagion.

It is worth outlining that the present study considered young adults aged between 18 and 34 years old. This choice was made consistently with studies that focused on the transition to adulthood in the Italian context, amidst the current unemployment rate and labor market’s insecurity for new entries in the transition from education to work. This appears to prolong young people’s family formation, which defines the ultimate entrance into adulthood in family-oriented countries such as Italy [91,92]. Nevertheless, future research could further investigate our results by referring to the theoretical proposal of Arnett [93], who, in the context of industrialized societies, introduced emerging adulthood to conceptualize the life phase ranging from late teens to mid-to-late 20s.

Another limitation of this study was the prevalence of females among the participants. The gender of participants was controlled in the statistical analysis carried out in the current study, but we did not specifically verify the presence of gender effects. Therefore, future studies should recruit more male participants and test the potential effects of gender on the associations and mediations observed, considering also the extant literature that has acknowledged women’s higher levels of death anxiety compared to men, a result that has also been confirmed during the COVID-19 pandemic [94].

Future research is warranted to replicate and extend these findings, overcoming the limits of the current study. In particular, longitudinal studies are necessary to collect data allowing for causal inferences. In addition, the prospective design can reduce some of the method bias concerns associated with gathering all data—predictor and criterion measures—simultaneously [88]. Future research could employ mixed-methods designs to reduce the risk of common method effects, combining self-report assessments with semi-structured interviews to be analyzed with qualitative methods. In addition, our results could be deepened by employing in-depth qualitative methods to be analyzed with reflexive thematic analysis [95] to develop an understanding of the core meanings attributed by young adults to the pandemic and their perceived influence of fear of death on their psychological well-being.

Furthermore, future research should control for and analyze the impact of actual life-threatening events and losses on the variables investigated. Severe chronic medical conditions within the family, which can be experienced by young adults as ambiguous loss or anticipatory grief [96,97,98], and different experiences of grief and mourning [99,100] should also be considered. 

In addition, future research should also consider an alternative mediating model, testing the potential mediation role of fear of death in the relationship between psychological inflexibility and eudaimonic well-being.

Lastly, following a life cycle perspective of emotion regulation [101], it will be useful to extend the focus to previous developmental ages, such as adolescence, and also consider how young adults turn to others to be helped or to help others in managing challenging emotions and fears. Indeed, the field of study concerning interpersonal emotion regulation is currently growing in importance [102] and can add new intriguing perspectives to the knowledge of how young adults regulate their emotions in challenging periods like the pandemic.

## 5. Conclusions

Despite the limitations, the current study is important for several reasons. It corroborates the importance of considering psychological inflexibility as a mechanism affecting individuals’ eudaimonic well-being, both in everyday life and in critical periods such as the pandemic. A growing body of literature suggests that psychological flexibility can be successfully improved through interventions targeting personal and interpersonal skills [47] and mindfulness/meditation programs [103,104], promoting various health outcomes [105]. 

Moreover, the study for the first time proposes a possible negative role of fear of death in influencing several domains of eudaimonic well-being of young adults, via the mediating link of psychological inflexibility. While these findings need to be validated through future research, they nonetheless highlight the significance of measures concerning fear and death anxiety in the psychological assessment of young adults, assessments that should be conducted by experienced, well-trained, or supervised health care professionals, capable of reliably administering the psychological instruments while promoting the engagement of the young person [106,107]. At the same time, our results suggest the importance of psychologically sustaining young adults in the uncertain and existentially challenging aftermath of the COVID-19 outbreak through appropriate psychological interventions also targeting their fears and anxiety of death, aiming to increase their psychological well-being.

## Figures and Tables

**Table 1:** Descriptive statistics and Pearson’s correlations among measures (*n* = 317).

Measure	M	SD	1	2	3	4	5	6	7	8	9
1. Autonomy (PWB)	38.34	8.23	–	0.40 **	0.40 **	0.26 **	0.32 **	0.40 **	−0.42 **	−0.17 **	−0.26 **
2. Environmental mastery (PWB)	35.03	9.31		–	0.54 **	0.40 **	0.76 **	0.75 **	−0.65 **	−0.21 **	−0.25 **
3. Personal growth (PWB)	46.10	6.34			–	0.39 **	0.54 **	0.63 **	−0.43 **	−0.07	−0.05
4. Positive relations with others (PWB)	38.91	9.97				–	0.38 **	0.50 **	−0.47 **	0.03	−0.05
5. Purpose in life (PWB)	36.97	9.43					–	0.72 **	−0.62 **	−0.19 **	−0.20 **
6. Self-acceptance (PWB)	36.23	9.11						–	−0.74 **	−0.13 *	−0.19 **
7. Psychological inflexibility (AAQ-II)	23.47	10.14							–	0.26 **	0.30 **
8. Fear of death and dying of self (CL-FODS)	43.64	12.52								–	0.57 **
9. Fear of death and dying of others (CL-FODS)	53.14	9.84									–

Note. M: mean; SD: standard deviation; * *p* < 0.05; ** *p* < 0.01.

## Data Availability

The data presented in this study are available on request from the corresponding author.
